# Punishment Sensitivity Predicts the Impact of Punishment on Cognitive Control

**DOI:** 10.1371/journal.pone.0074106

**Published:** 2013-09-13

**Authors:** Senne Braem, Wout Duthoo, Wim Notebaert

**Affiliations:** Department of Experimental Psychology, Ghent University, Ghent, Belgium; Ecole Normale Supérieure, France

## Abstract

Cognitive control theories predict enhanced conflict adaptation after punishment. However, no such effect was found in previous work. In the present study, we demonstrate in a flanker task how behavioural adjustments following punishment signals are highly dependent on punishment sensitivity (as measured by the Behavioural Inhibition System (BIS) scale): Whereas low punishment-sensitive participants do show increased conflict adaptation after punishment, high punishment-sensitive participants show no such modulation. Interestingly, participants with a high punishment-sensitivity showed an overall reaction time increase after punishments. Our results stress the role of individual differences in explaining motivational modulations of cognitive control.

## Introduction

The subject of cognitive control, the ability to monitor our environment and adapt to ever-changing contexts, has been of increasing interest to psychologists over the past decades. However, the interactions between cognitive-control mechanisms and motivational variables are not well understood. Previous studies have demonstrated how overall conflict processing [1], as well as adaptations to conflict [2,3], can be enhanced after reward. While we have these first notions on the role of reward in modulating cognitive control, the influence of punishment on conflict adaptation remains unclear. However, we encounter negative feedback all the time (e.g., annoying computer beeps indicating wrong key presses) and it is important to understand how these signals interact with cognitive processes. In the present study, we set out to investigate if, how, and when, punishment can modulate trial-to-trial adaptations to cognitive conflict.

In research on cognitive control, conflict tasks are typically used. In the flanker task [4], for example, in which the central target is presented together with either congruent (> > >) or incongruent (> < >) flankers, participants respond faster and more accurately on congruent trials compared to incongruent trials (i.e., the flanker effect). Interestingly, Gratton, Coles, and Donchin [5] observed that there was a smaller flanker effect after incongruent than after congruent trials (termed the conflict adaptation or Gratton effect). There are a number of different frameworks dealing with this Gratton effect and we will discuss three of the most prominent theoretical models. These models all differ in describing the exact mechanics for explaining the Gratton effect, and the role of motivational variables there-in. Interestingly, however, all three models predict a similar modulation by punishment.

According to the Conflict Monitoring Theory (CMT) [6,7], conflict, as an aversive signal [8-10], is detected by the anterior cingulate cortex (ACC) which triggers subsequent behavioural adaptation by enhancing focus to task-relevant information, implemented by the dorsolateral prefrontal cortex. The CMT suggests that the aversiveness of cognitive conflict is what drives conflict adaptation [6]. In support of this view, stressing the importance of negative valence in bringing about adaptations to conflict, van Steenbergen, Band, and Hommel [11-14] demonstrated how positive affect can counteract the Gratton effect. Therefore, a similar but aversive teaching signal would add to this signal [11-14] and thereby enhance conflict adaptation, resulting in a more pronounced Gratton effect (see also 15).

As an alternative account of cognitive control, the adaptation-by-binding account (ABBA) of Verguts and Notebaert [16] suggests that conflict strengthens currently active connections. In this model, an arousal signal is sent throughout the brain upon the detection of cognitive conflict [17], which strengthens, through Hebbian learning, all active task-relevant connections. By binding these associations after conflict, a smaller congruency effect will occur on the following trial, reflected in the Gratton effect [16,18]. The ABBA predicts that arousing stimuli, irrespective of their valence, would help to increase adaptations following conflict [16]. Therefore, this account also hypothesizes that punishment signals would enhance the Gratton effect.

While both the CMT and ABBA focus on reactive control (adjusting information processing after the detection of conflict), Braver, Gray and Burgess [19] make a distinction between proactive and reactive modes of cognitive control (Dual Mechanisms of Control or DMC, see also 20). Whereas proactive control refers to a more anticipatory mode of cognitive control, where priorities are set before the occurrence of the cognitive conflict, reactive control refers to the mode of control driven by situational events, for example, the trial-by-trial adaptations to cognitive conflict as described above. This dissociation is important, because the DMC predicts that the proactive and reactive modes of cognitive control are differentially affected by motivational variables. Specifically, the DMC framework predicts that rewards promote proactive control, while punishments enhance reactive control [20-22]. Therefore, conceptualizing the Gratton effect as a manifestation of reactive control, we can again predict that punishments will enhance adaptations to conflict.

However, although all three models seem to predict a similar modulation by punishment, a first study investigating the modulation of the Gratton effect by punishment signals [3] showed no modulation, whereas reward signals effectively enhanced the Gratton effect [3] (see also 2). Interestingly, Stürmer, Nigbur, Schacht, and Sommer [3] observed an overall reaction time increase after punishments, rather than increased conflict adaptation. This suggests that punishment distracted participants from the task, similar to what happens after participants make an error [23,24]. Consequently, it is possible that punishments are perceived as too salient, slowing down subsequent performance rather than increasing task focus.

As a first test of this hypothesis, we take individual differences in sensitivity to punishment into account. As recently stressed in a review on the influence of emotion and motivation on cognitive conflict [25], individual differences in sensitivity to emotional or motivational stimuli have a major impact on how such signals modulate conflict processing. Therefore, we hypothesized that the influence of punishment on performance should vary as a function of punishment sensitivity. In order to assess punishment sensitivity in the present study, we administered the BIS- (Behavioural Inhibition System) and BAS-scales (Behavioural Activation System [26]), which have proven to be valuable tools in predicting how individual differences in punishment or reward sensitivity can modulate motivational effects on cognition (e.g., [2,11,27-32]). We predicted that increased cognitive control following punishment would be restricted to participants that are not highly sensitive to punishment, while high punishment-sensitive participants will probably not benefit or learn from punishments. Highly punishment-sensitive people (scoring high on BIS) perceive punishments as more aversive [29] and are more likely to be distracted by punishments [33].

To this end, we opted to use exactly the same design as in our reward study [2], in which a four-choice colour flanker experiment was combined with reward signals on 25% of the trials. Yet, instead of these rewards, we now presented punishments. Participants were informed that 25% of the trials could be punished. This was to keep punishment expectations similar across participants. Yet, when participants responded very fast and accurate, a punishment could be avoided. This implementation of punishments ensures that punishment signals are randomly distributed, but still performance-contingent. In our opinion, this schedule is preferable to a punishment schedule where the 25% slowest responses are punished (as used in [3]). Specifically, in the design of Stürmer and colleagues [3], an adaptive algorithm was employed to closely monitor participants’ behaviour, allowing the experimenters to punish the 25% slowest responses per participant. However, because only the slowest trials were punished, trials following punishment were confounded with trials following slow performance, making it hard to disentangle the effects of previous punishment presentation versus previous task performance. In contrast, the punishment signals in our design were randomly distributed (but still performance related), allowing us to investigate the direct effects of punishment presentation only.

Furthermore, we chose to present punishment signals without an inherent affective value (we presented "-1", denoting the loss of a point in the participants’ score). This was to ensure that we were investigating the modulation of cognitive control by punishment, rather than negative affect alone. For example, when presenting a negative smiley or picture as a punishment signal, our effect of punishment could be confounded with an effect of negative affect that is not punishment-induced. As argued in our previous reward study [2], affective and reinforcement signal modulations of cognitive control should be distinguished from one another (see also 34-36).

In sum, we administered a flanker task with performance-contingent punishment signals and verified whether punishment sensitivity, as measured by the BIS-scale, modulated punishment-induced modulations of cognitive control.

## Methods

### Ethic Statement

The study was approved by the ethical committee of the Faculty of Psychology and Educational Sciences of Ghent University. All participants signed an informed consent prior to the experiment.

### Participants

Twenty-six students took part in return for credits or 6€ (range = 18-21 years, 22 female, 22 right-handed) based on their written informed consent with approval of the local ethical committee and according to the Declaration of Helsinki.

### Stimuli and Material

The flanker stimuli consisted of three horizontally aligned, centrally presented squares that were printed in one of the four possible colours (green, yellow, blue or red). Both flankers had either the same (congruent, e.g., red-red-red) or a different (incongruent, e.g., blue-red-blue) colour than the central square. The stimuli were presented on a Pentium, with the use of Tscope software [37]. A hand response box was used to register the responses.

### Procedure and Design

Participants were asked to respond to the colour of the central square by pressing one of the four horizontally aligned response buttons, using their index and middle fingers. Subjects were randomly assigned to one of four response mappings, which were created by shifting the colour-to-button assignment. After a practice block of 48 trials, participants performed 14 experimental blocks of 48 trials. For each experimental block, an equal number of congruent and incongruent trials were presented in a random order: 25% of the trials were punishment trials, which were randomized for the congruent and incongruent trials separately. Between blocks, participants were allowed a self-paced break during which they could see their updated score. For every ten participants, the participant with the best score received a store coupon worth 10€. Each subject started with a score of 300 points and could only lose points on punishment trials, as indicated by feedback presentation in the form of "-1". Hence, whenever seeing "-1" participants knew that they just lowered their chances of winning a store coupon. There was no option to gain points. All participants were truthfully instructed about this reinforcement schedule.

First, a fixation cross was presented for 500 milliseconds, after which the target and flanker stimuli were presented and remained on the screen until the participant responded. The maximum response time was 1000 milliseconds. On punishment trials, the participant was given feedback in the form of “-1” centrally presented on the screen for 500 milliseconds, unless he or she responded correctly faster than 350 milliseconds. In the latter case, or after a “no-punishment” trial, a blank screen was presented for 500 milliseconds. Finally, a blank screen was presented for 1000 milliseconds, whereupon the next trial started.

Although participants knew from the instructions that the punishment signals were performance-contingent, we wanted to ensure that participants also experienced those as such. In the reward version of this experiment [2], people missed out on their reward on 10% of the potential rewarding trials. We believe that this 10% is enough for participants to feel that their reinforcement signals are performance-contingent. Therefore, we aimed at enabling participants to escape their punishments in 10% of the trials. In this light, the abovementioned 350 milliseconds deadline was chosen, because earlier versions of this experiment [2] showed that only 10% of the responses were faster than 350 milliseconds. Indeed, also in the present experiment, participants were faster than 350 milliseconds on only 10.8% of the trials.

### Questionnaires

Immediately after the experiment, participants completed the Behavioural Inhibition System/Behavioural Activation System (BIS/BAS) Scales [26]. This took the form of 20 questions, such as "I feel worried when I think I have done poorly at something important" and "When I get something I want, I feel excited and energized", respectively examining punishment and reward sensitivity. Seven items score punishment sensitivity, averaged into a BIS-score, for which higher values indicate higher punishment sensitivity. The thirteen remaining items all score reward sensitivity (BAS-score), sometimes divided into its three subscales: BAS Reward Responsiveness (5 items), BAS Drive (4 items), and BAS Fun (4 items). A higher value on these scales indicates a higher form of reward sensitivity.

## Results

One participant was excluded from the analysis because of a mean accuracy (= 0.45) two standard deviations below the group average (*M* = 0.76; *SD* = 0.12). Trials following an error and the first trial of each block were removed from further analyses (24.5% of the trials). Also, trials following a trial where the response time (RT) was faster than 350 milliseconds were also excluded (another 8.4% of the remaining trials) to ensure that the effect of previous feedback was not confounded with previous RT (trials faster than 350 milliseconds were never punished). For the RT analyses, errors were also excluded (13.2% of the remaining trials) and from these remaining trials, RT outliers (± 2*SD* of the mean reaction time calculated per subject and per condition) were removed (2.2%). This means that a total of 48.3% of the trials were excluded for the RT analyses, primarily due to the relatively high error rate (24%) in this experiment. This observed error rate is clearly higher than the reward version of our experiment (10% [2]). Conversely, reaction times were substantially faster (480 ms) in our punishment experiment, as compared to the reward experiment (530 ms). We believe that this main difference in task performance represents an important dissociation in response strategy elicited by the different reward and punishment conditions (see also, 22,38). Specifically, punishments could only be avoided by responding faster than 350 milliseconds (and accurately), inducing a speeded response strategy, while rewards in [2] could be obtained by responding accurately (and faster than 1000 milliseconds), promoting a more accurate response strategy. However, excluding this high number of trials did not influence our main findings (removing the two participants with less than ten data points in one condition from the analyses did not change the significance of our analyses).

Next, we carried out an ANOVA with three within-subject factors (congruency, previous congruency and previous feedback), with RTs and error rates as dependent variables. We observed a significant congruency effect, *F*(1,24) = 77.025, *p* < .001, which interacted with previous congruency, *F*(1,24) = 19.686, *p* < .001, indicating a significant overall Gratton effect of 23 ms (as calculated by subtracting the congruency effect after incongruent trials from the congruency effect after congruent trials). Although the Gratton effect after punishments was numerically larger than the Gratton effect after no-punishment trials (31 vs. 15 ms, respectively), this modulation did not reach significance, *F*(1,24) = 2.188, *p* > .1. The error rates only showed a significant congruency effect, *F*(1,24) = 14.698, *p* < .01. It could be argued that the feedback presentation on punishment trials (versus blank screen on no-punishment trials) in our experiment might have counteracted an overall modulation of the Gratton effect. However, our findings are in line with the study of [3], who did present feedback after neutral trials. Furthermore, in our previous study [2], we ran two control studies that demonstrated how infrequent irrelevant stimulus presentations (vs. no visual stimulation) during the inter-trial interval did not modulate adaptations to conflict.

**Figure 1:**
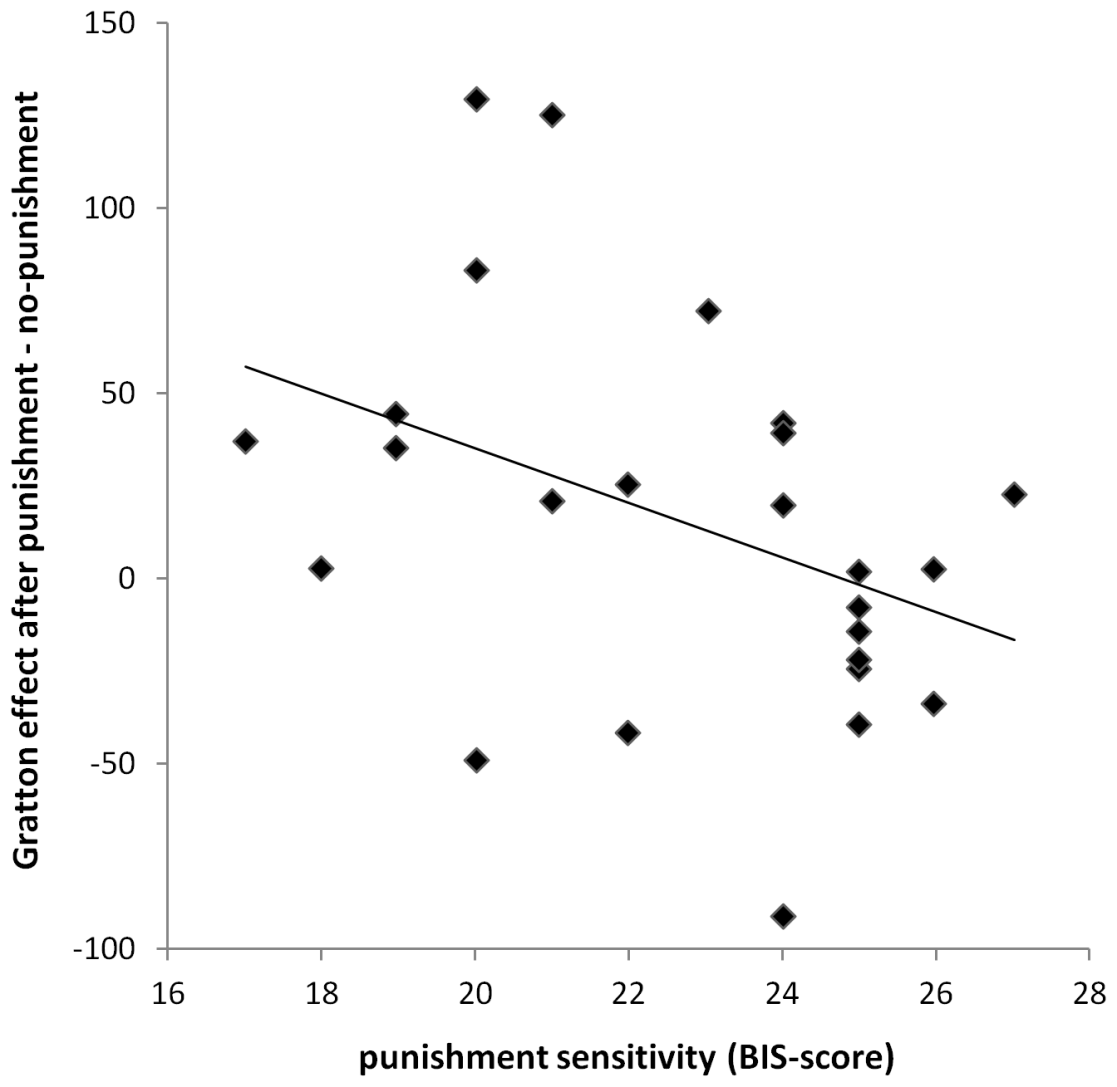
The scatter plot shows the correlation between individual scores on the BIS scale and the difference scores for the Gratton effects.

Next, we wanted to investigate how individual differences in punishment sensitivity (as measured by the BIS-scale) modulate the effect of punishment on conflict adaptation. To this end, we ran a hierarchical linear regression analysis in which the Gratton effect served as a dependent variable. In a first step, previous feedback, and the standardized BIS and BAS scores were entered, and in a second step, the interaction between BIS and previous feedback was included. As expected, the three variables BIS, BAS, and previous feedback (Model 1) did not significantly contribute to the regression model of the Gratton effect. The model accounts for R^2^ = 8.2 %, *F*(3,46) = 1.365, *p* > .1. Adding the interaction variable (Model 2), we observed a significant ΔR^2^ of 7.7%, Δ*F*(1,45) = 4.126, *p* < .05, denoting that the interaction between BIS and previous feedback was a significant predictor of the Gratton effect, after having controlled for the effects of BAS, BIS, and previous feedback. This relation between BIS, previous feedback and the Gratton effect is depicted in Figure 1, where the correlation between the modulation of the Gratton effect (as calculated by subtracting the Gratton effect after no-punishment trials from the Gratton effect after punishment trials) and participants’ punishment sensitivity is plotted (Spearman’s rho correlation, ρ = -.455, *p* < .05). No Spearman’s rho correlations were observed with the BAS-scale, or any of its subscales.

To further investigate the role of punishment sensitivity in the modulation of the Gratton effect, we decided to split up our subjects in a low and high punishment-sensitive group by means of a median-split analysis on participants’ BIS-score: participants with a BIS-score lower than 24 were assigned to the low punishment-sensitive group and participants with a BIS-score of 24 or higher were assigned to the high punishment-sensitive group. Note that the mean BIS-score (20.2) for the low punishment-sensitive group is actually similar to the average BIS-scores collected from large community samples, which are typically between 19 and 22 [39,40]. The mean BIS-score (25) for the high punishment-sensitive group on the other hand, can be considered relatively high. Next, we re-analysed our data with punishment sensitivity as a between-subjects factor.

In line with the observed correlation, we found a four-way interaction between congruency, previous congruency, previous feedback, and punishment sensitivity in reaction times, *F*(1,23) = 6.680, *p* < .05. Interestingly, the error rates showed a similar trend, *F*(1,23) = 3.253, *p* = .084. No other effects of punishment sensitivity were observed (all ps > .1).

**Figure 2:**
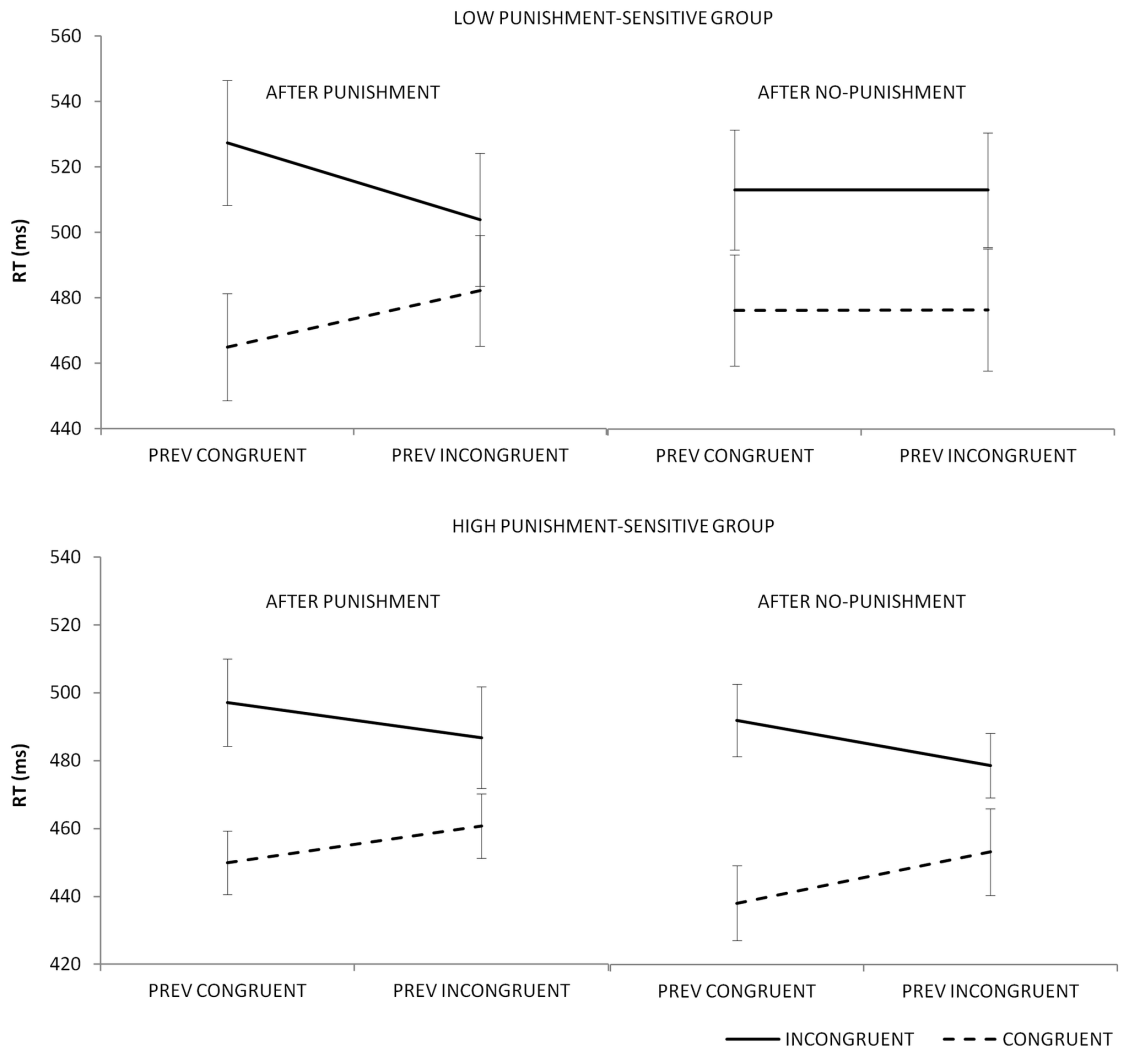
The reaction times for each punishment-sensitive group separately. The figure demonstrates how punishment helps in adapting to conflict, for low punishment-sensitive people, while people high in punishment sensitivity slow down after punishment. The error bars are ± 1 standard error.

In order to gain further insight in these behavioural differences between both punishment sensitivity groups, we conducted ANOVAs for each punishment sensitivity group separately. As depicted in Figure 2, the low punishment-sensitive group showed a modulation of the Gratton effect, *F*(1,11) = 6.269, *p* < .05: the Gratton effect after punishment trials (41 ms) was more pronounced than the Gratton effect after no-punishment trials (0 ms; this absence of a Gratton effect after no-punishment trials will be discussed in the general discussion). The high punishment-sensitive group, on the contrary, showed an overall significant Gratton effect, *F*(1,12) = 10.511, *p* < .01, that was not modulated by previous feedback, *F*(1,12) < 1. However, a main effect of previous feedback indicated that the high punishment-sensitive group did show a general slowing after punishments, *F*(1,12) = 11.443, *p* < .01, whereas the low punishment-sensitive group did not, *F*(1,11) = 0.004, *p* > .1. The modulation of the Gratton effect per punishment-sensitivity group in the error rates, as well as each Gratton effect separately, garnered no significant results in both conditions, but followed a numerically similar trend as the reaction times, as summarized in Table 1.

**Table 1:** Mean error rate (%) as a function of previous congruency, current congruency and preceding feedback for each punishment sensitivity group differently.

		*low punishment-sensitive group*			*high punishment-sensitive group*	
*n*-1	*n*	*after punishment*	*after no-punishment*		*after punishment*	*after no-punishment*
C	C	16.8	19.5		18.5	15.0
	I	25.4	23.3		21.9	22.1
I	C	21.6	19.8		13.6	17.3
	I	22.9	23.4		24.4	21.5

Note: C = congruent; I = incongruent; *n* - 1 = preceding trial; *n* = current trial.

Alternatively, instead of exploring the four-way interaction between congruency, previous congruency, previous feedback, and punishment sensitivity, by looking at the separate punishment-sensitivity groups, we also performed analyses on the effects of punishment sensitivity on the Gratton effect, after punishment and no-punishment trials separately. The Gratton effect after punishment was not significantly larger in the low punishment-sensitive than in the high punishment-sensitive group, *F*(1,23) = 1.275, *p* > .1. After no-punishment trials, however, the Gratton effect was significantly smaller in the low punishment-sensitive group than in the high punishment-sensitive group, *F*(1,23) = 8.991, *p* < .01. This interaction hints at differences in the respective Gratton effects. Therefore, we tested the Gratton effects in all four conditions separately. The Gratton effect after punishment reached marginal significance, *F*(1,12) = 3.413, *p* = .089, in the high punishment-sensitive group (21 ms), and significance, *F*(1,11) = 9.459, *p* < .05, in the low punishment-sensitive group (41 ms). The Gratton effect after no-punishment trials in the high punishment-sensitive group (29 ms) was also significant, *F*(1,12) = 21.516, *p* < .01, while this Gratton effect in the low punishment-sensitive group (0 ms) was not, *F*(1,11) = 0.001, *p* > .1.

## Discussion

In line with previous studies [3], a group analysis of the current study suggested that punishment signals do not influence adaptations to conflict. However, by taking into account individual differences in punishment sensitivity, we have demonstrated how punishment can have an effect on cognitive control, depending on the punishment’s perceived severity. Participants low in punishment-sensitivity showed an enhanced Gratton effect after punished trials. Participants high in punishment-sensitivity showed no such modulation. Instead, highly punishment-sensitive participants slowed down after punishments.

Our results clearly stress the importance of taking into account individual differences when studying the role of motivational variables in modulating cognitive control [25]. Especially when investigating motivational or emotional influences on cognitive control, individual differences in sensitivity can help expose underlying mechanisms of how these variables influence cognitive adaptations (e.g., [2,12,27-32]). For example, using electrophysiological recordings, De Pascalis, Varriale, and D’Antuono [31] demonstrated a higher feedback-related negativity following punishments as a function of participants’ BIS-score. Boksem and colleagues [29] investigated the influence of punishment sensitivity (as measured by the BIS-scale) on behavioural adaptations after punished performance errors and demonstrated how high punishment-sensitive people show more slowing after punishments than low punishment-sensitive people do. Our results add to these findings by demonstrating that punishments can also promote adaptive behaviour, as long as people are not too sensitive to punishments.

Surprisingly, in the low punishment-sensitive group, the modulation of the Gratton effect was not only reflected in a larger Gratton effect after punishments, but also an absent Gratton effect after no-punishment trials. This finding is in line with earlier studies investigating motivational effects on cognitive control, in that these studies also demonstrated how the motivationally less significant condition is not the neutral and constant baseline as sometimes assumed (e.g., [2,41-43]). Instead, by introducing a motivationally significant reinforcement signal, a context is created where both punishment and no-punishment trials receive an informative value: by increasing the motivational value of punishment trials the value of no-punishment trials is simultaneously decreased.

At first sight, our findings might sound counterintuitive in suggesting that higher sensitivity to a reinforcement signal is associated with lower benefits of this reinforcement signal on task-adaptive behavior. Instead, task-performance seems to be impeded, rather than promoted after punishment for high punishment-sensitive people. Therefore, we believe our results might reflect the right hand side of an inverted-U shaped function between punishment saliency and task performance. Too often, existing theories of cognitive psychology assume a linear, more-is-better effect of cognitive variables (e.g., medication, reward, working memory capacity, etc.) on performance, whereas both empirical progress [44] and evolution theory [45] have indicated it is wise to assume a curvilinear, inverted U-shape function. Our findings corroborate this idea: by taking into account punishment sensitivity, we demonstrated how we are able to flexibly adapt our behaviour after punishments, as long as they do not overwhelm us.

Specifically, our findings could be framed within the Yerkes-Dodson law [46], which suggests that there is an inverted U-shaped relation between arousal and task performance. Reinforcement signals that are too arousing will decrease, rather than increase, task performance. We can interpret our data as an extension of the Yerkes-Dodson law, in the sense that not only general task performance is modulated by a curvilinear function of arousal, but also cognitive control is (i.e., trial-to-trial adaptations to conflict). This, however, does not mean that our findings can exclusively be explained in terms of the ABBA, which describes adaptations to conflict as a function of (conflict-induced) arousal [16]. In fact, the arousing value of a punishment might as well directly impact the experienced aversiveness [6] or the saliency with which it will promote reactive control [20].

We suggest that for the low punishment-sensitive group of participants that showed an enhanced Gratton effect following punishment signals, punishments induced the appropriate levels of saliency for increasing cognitive control. This modulation could reflect an enhanced strengthening of task relevant associations [16,18], a modulation of task attention [6,7], or a shift from a more proactive to a more reactive control modus [20,21], after punishment. However, participants highly sensitive to punishment showed no modulation of the Gratton effect after punishment, suggesting that arousal levels were too high to modulate cognitive control processes. These participants showed an overall increase in response latencies following punishment. This finding is in line with [3]. Similarly, Padmala, Bauer, and Pessoa [47] showed how arbitrarily presenting highly arousing negative pictures (i.e., pictures of mutilated corpses) in between Stroop trials, elicited an overall reaction time slowing, and even a reduced conflict adaptation effect.

A similar inverted U-shaped relationship between aversiveness and performance has also been proposed in the literature investigating block-wise manipulations of conflict adaptation. For example, while van Steenbergen and colleagues [12,13] demonstrated how conflict adaptation can be increased under a negative mood manipulation (either music/imagination- or medicine-induced), Meiran, Diamond, Todor, and Nemets [48] showed how individuals with major depressive disorder show decreased, rather than increased, conflict adaptation. In reviewing these discrepancies in results, van Steenbergen and colleagues [13] concluded that negative mood can help promoting adaptations to conflict, but only up to a certain level. When being experienced as too high to cope with, negative mood can lead people to disengage from the task, resulting in decreased conflict adaptation [48]. This is also consistent with the mood-behavior model of Gendolla [49] which states that the relation between mood and effort mobilization is nonlinear.

The overall differential influence of punishment and reward signals, in that reward signals modulate adaptations to conflict [3,2] while punishment signals do not ( [3] and our overall analysis), could also be attributed to a difference in arousal levels induced by both reinforcement signal types. For example, keeping everything else equal, Gomez and McLaren [38] systematically examined the effects of reward and punishment signals and demonstrated how punishment schedules (as opposed to reward schedules) induced higher overall arousal levels, as measured by the skin conductance response. Similarly, comparing appetitive with aversive motivational systems, Tranel [50] demonstrated how the latter was associated with an increased skin conductance response, while the former was not. These findings are also consistent with the more general idea of a negativity bias, which relates to the finding that people tend to pay more attention and give more weight to negative, as compared to positive experiences [51,52].

In fact, this apparent different impact of rewards and punishments may reflect a similar difference observed between post-conflict and post-error behavioural adjustments. While cognitive conflict seems to help task focus, errors elicit an orienting response, causing an overall slowing rather than enhanced performance [53]. Interestingly, both post-error and post-conflict processes have been linked to arousal (e.g., [3,17,24,54,55]), yet conflict-induced arousal, although reliable, seems to be substantially smaller than error-induced arousal [17]. Similar to error processing, we suggest that people highly sensitive to punishment may have experienced a short-lived orienting response [23,24] towards the punishment signal, but away from the task, reflecting a failure to disengage from the punishment. Analogously, it has been demonstrated that high punishment-sensitive people attend longer to aversive stimuli and have difficulty disengaging attention from these stimuli [33].
